# Outage Performance of SWIPT-D2D-Based Hybrid Satellite–Terrestrial Networks

**DOI:** 10.3390/s25082393

**Published:** 2025-04-09

**Authors:** Zhen Li, Jian Xing, Jinhui Hu

**Affiliations:** College of Computer and Control Engineering, Northeast Forestry University, Harbin 150040, China; lizhen5301@126.com (Z.L.); hujinhui0213@163.com (J.H.)

**Keywords:** simultaneous wireless information and power transfer, internet of things, hybrid satellite–terrestrial networks, device-to-device, outage probability

## Abstract

This paper investigates the outage performance of simultaneous wireless information and power transfer (SWIPT)-assisted device-to-device (D2D)-based hybrid satellite–terrestrial networks (HSTNs). In the considered system, an energy-constrained terrestrial user terminal (UT) harvests energy from the radio frequency (RF) signal of a terrestrial amplify-and-forward (AF) relay and utilizes the harvested energy to cooperate with the shadowed terrestrial Internet of Things (IoT) devices in a D2D communication. Both power splitting (PS)-based and time switching (TS)-based SWIPT-D2D schemes are adopted by the energy-constrained UT to obtain sustainable energy for transmitting information to the shadowed IoT device. Considering shadowed Rician fading for satellite–terrestrial links and Nakagami-*m* fading for terrestrial links, we analyze the system performance by deriving the closed-form expressions for the outage probability (OP) of both the UT and the IoT device. Our theoretical analyses are validated via Monte Carlo simulations.

## 1. Introduction

With the rapid development of communication networks, the application scope of the Internet of Things (IoT) has become increasingly extensive and diversified, and the traffic of mobile data services has also witnessed explosive growth. In general, the deployment of fifth-generation (5G) communication networks has further promoted the development of the IoT [1]. However, considering the limitations of infrastructure costs and geographical environment factors, terrestrial networks are mainly deployed in developed regions and densely populated urban areas, and are unable to cover the vast airspace, sea area, etc. This has significantly restricted the deployment and application scope of IoT devices. Therefore, the aspiration of this work is to formulate the design of sixth-generation (6G) communication networks, which enable ubiquitous communication [2,3,4].

Considering that satellite communication systems can provide global users with high-speed broadband access without geographical constraints, the architecture of hybrid satellite–terrestrial networks (HSTNs), formed by integrating terrestrial networks and satellite communication systems, can meet the requirements of the 6G communication networks to achieve ubiquitous coverage [5,6,7]. The HSTN framework is proposed in reference [8], which examines the role of the satellite communication system within HSTNs and outlines its future development directions.

In the context of HSTNs, most research studies in the open literature have focused on such networks adopting amplify-and-forward (AF) and decode-and-forward (DF) relaying protocols. In reference [9], the AF relaying HSTNs with a masked destination node have been analyzed over shadowed Rician fading and Nakagami-*m* fading channels. The performance of the multiuser AF relaying HSTNs with opportunistic scheduling has been investigated in reference [10]. Reference [11] analyzes the outage performance of the non-orthogonal multiple access (NOMA)-based DF relaying HSTNs. Reference [12] studies the physical-layer security of the HSTNs composed of multiple DF relays and users in the presence of a terrestrial eavesdropper. Therefore, the terrestrial relaying network can effectively assist HSTNs in addressing the shadowing effect caused by obstacles, weather factors, angular differences, and other factors.

However, considering the situation that the IoT devices affected by the shadowing effect are outside the coverage area of the terrestrial relay, the cooperation of the terrestrial relay will no longer be available in the downlink transmission of the HSTNs. In order to achieve the effective transmission of information between the satellite and the shadowed IoT devices, cooperative communication can be realized by forming a device-to-device (D2D) communication architecture with a neighboring user terminal (UT). However, considering that most UTs are energy-constrained in practical scenarios, it is unsustainable for the UTs serving as D2D transmitters to continuously provide cooperation for the shadowed IoT devices over an extended period.

To address the issue of energy limitation, we consider introducing simultaneous wireless information and power transfer (SWIPT), which is proposed in reference [13], to extend the lifetime of the D2D communication. Without affecting the information decoding (ID), the energy-constrained UT harvests energy from a portion of the received radio frequency (RF) signal to deliver information to the D2D receiver. Both power splitting (PS) and time switching (TS) receiver architectures are proposed in reference [14]. By adopting PS-SWIPT, the UT splits the received signal into two portions, with one for energy harvesting (EH) and the other for ID [15,16]. By adopting TS-SWIPT, the UT switches in time between EH and ID [17,18,19]. Reference [20] investigates the performance of a two-way DF relay network over Nakagami-*m* fading channels adopting PS and TS schemes. Reference [21] investigates the performance of a two-hop AF MIMO relay network with an energy-constrained relay node adopting TS schemes. However, most existing literature around SWIPT schemes is focused on the performance of terrestrial relaying networks, and little considers the scenario of heterogeneous HTSNs.

Motivated by the above, we consider the HSTNs in which an energy-constrained terrestrial UT cooperates with a shadowed terrestrial IoT device in a D2D communication. Both PS-based and TS-based SWIPT-D2D schemes are adopted by the energy-constrained UT to obtain sustainable energy for delivering information to the shadowed IoT device. We investigate the impact of the proposed SWIPT-D2D schemes by deriving the closed-form expressions for the outage probability (OP) of both the UT and the IoT device. The main contributions of this paper are summarized as follows: (1) we propose the framework of the HSTNs with PS-D2D and TS-D2D schemes; (2) we characterize the probability density function (PDF) and cumulative distribution function (CDF) of the signal-to-noise ratios (SNRs) over the hybrid satellite–terrestrial links; (3) we derive the closed-form expressions for the OP by adopting the PS-D2D and TS-D2D schemes.

The rest of this paper is organized as follows: The PS-D2D-based and TS-D2D-based HSTN models are presented in Section 2. The theoretical derivation of the OP expressions is investigated in Section 3. Monte Carlo simulations are provided in Section 4. The conclusion is given in Section 5.

## 2. System Model and SWIPT-D2D Schemes

**Figure 1:**
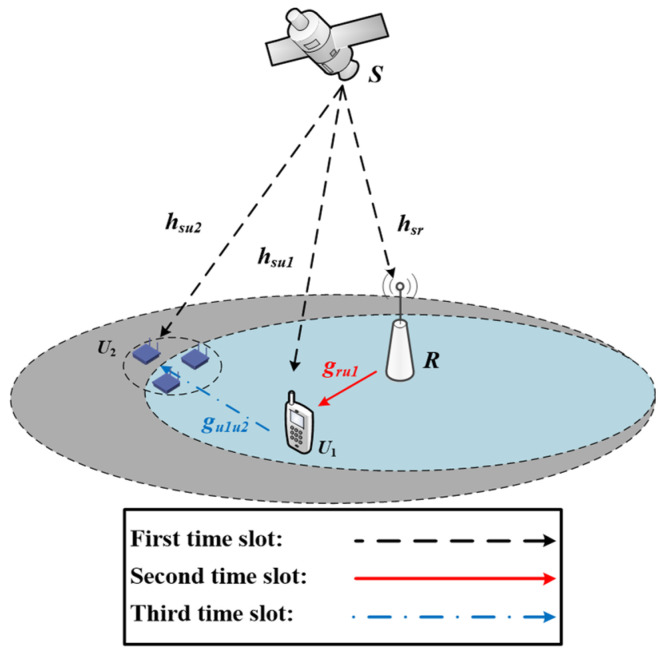
Illustration of the PS-D2D-based HSTN model.

Let us consider the PS-D2D-based HSTNs, in which a low earth orbit (LEO) satellite *S* broadcasts information to an energy-constrained UT *U*_1_ and a shadowed IoT device *U*_2_ with the assistance of a terrestrial AF relay *R*, as shown in Figure 1. The location of the IoT device *U*_2_ is outside the coverage of relay node *R*, and it cannot completely receive the information sent by satellite source node *S* due to factors such as the shielding effect. Thus, it is necessary to complete the information transmission from satellite *S* to IoT device *U*_2_ with the cooperation of UT *U*_1_. We assume that the satellite *S*, UT *U*_1_, and IoT device *U*_2_ are equipped with a single antenna and are operating in half-duplex mode.

In HSTNs, we assume that the channels between the satellite and the terrestrial nodes undergo independent shadowed Rician fading, which is commonly used in land mobile satellite (LMS) cooperative networks, and the channels among terrestrial nodes undergo independent Nakagami-*m* fading, which is commonly used in terrestrial communication networks. We also assume that the terrestrial nodes are inflicted by the additive white Gaussian noise (AWGN) with mean zero and variance $\sigma^{2}$.

### 2.1. PS-D2D Scheme

In the PS-D2D scheme, which is shown in Figure 1, we assume that *T* denotes the duration of the entire transmission process, which is divided into three orthogonal phases with equal time duration.

During the first time slot, with a time duration of *T*/3, the satellite *S* broadcasts its signal $x_{s}$ to the terrestrial nodes. Therefore, the received symbols at the terrestrial nodes can be respectively given by

(1) $$y_{si,ps}=\sqrt{P_{s}}h_{si}x_{s}+n_{si},$$

where $i\in \left\{r,u1,u2\right\}$, $h_{si}$ denotes the channel coefficient between *S* and the terrestrial nodes, respectively, $n_{si}$ denotes the AWGN, respectively, and $P_{s}$ denotes the transmit power of *S*.

During the second time slot, *R* amplifies and forwards $y_{sr,ps}$ to *U*_1_; the received symbol will be

(2) $$y_{ru1,ps}=\sqrt{P_{r}}g_{ru1}\frac{y_{sr,ps}}{\sqrt{{\left|y_{sr,ps}\right|}^{2}}}+n_{ru1},$$

where $g_{ru1}$ denotes the channel coefficient between *R* and *U*_1_, $n_{ru1}$ denotes the AWGN, and $P_{r}$ denotes the transmit power of *R*. After receiving $y_{ru1,ps}$, *U*_1_ splits the symbol into two portions with PS coefficient $\rho \in \left(0,1\right)$, where the portion $\sqrt{\rho }y_{ru1,ps}$ is utilized in ID and the portion $\sqrt{1-\rho }y_{ru1,ps}$ is utilized in EH. Thus, the harvested energy at *U*_1_ can be given by

(3) $$E_{u1,ps}=\frac{\omega T\left(1-\rho \right)P_{r}{\left|g_{ru1}\right|}^{2}}{3},$$

where $\omega \in \left(0,1\right)$ denotes the energy conversion efficiency. The received symbol at *U*_1_ can be rewritten as

(4) $$y_{ru1,z}=\sqrt{\rho }\left(\sqrt{P_{r}}g_{ru1}\frac{y_{sr,ps}}{\sqrt{{\left|y_{sr,ps}\right|}^{2}}}+n_{ru1}\right)+n_{ru1,z},$$

where $n_{ru1,z}$ denotes the AWGN. The SNR of satellite–terrestrial direct link at *U*_1_ is given by

(5) $$\Lambda_{su1,ps}=\eta_{s}{\left|h_{su1}\right|}^{2},$$

where $\eta_{s}=P_{s}/\sigma^{2}$. The SNR of the terrestrial relay link at *U*_1_ is given by

(6) $$\Lambda_{sru1,ps}=\frac{\rho^{\prime }\Lambda_{sr,ps}\Lambda_{ru1,ps}}{\Lambda_{sr,ps}+\rho^{\prime }\Lambda_{ru1,ps}+1},$$

where $\Lambda_{sr,ps}=\eta_{s}{\left|h_{sr}\right|}^{2}$, $\Lambda_{ru1,ps}=\eta_{r}{\left|g_{ru1}\right|}^{2}$, $\eta_{r}=P_{r}/\sigma^{2}$, and $\rho^{\prime }=\rho /\left(\rho +1\right)$.

During the third time slot, *U*_1_ decodes and forwards the signal $x_{u1,ps}$ to *U*_2_ with the harvested energy. The transmit power at *U*_1_ will be given by

(7) $$P_{u1,ps}=\frac{3E_{u1,ps}}{T}=\omega \left(1-\rho \right)P_{r}{\left|g_{ru1}\right|}^{2}.$$

Thus, the symbol received at *U*_2_ is given by

(8) $$y_{u1u2,ps}=\sqrt{\omega (1-\rho )P_{r}{\left|g_{ru1}\right|}^{2}}g_{u1u2}x_{u1,ps}+n_{u1u2},$$

where $g_{u1u2}$ denotes the channel coefficient between *U*_1_ and *U*_2_, and $n_{u1u2}$ denotes the AWGN. The SNR of the satellite–terrestrial direct link at *U*_2_ is given by

(9) $$\Lambda_{su2,ps}=\eta_{s}{\left|h_{su2}\right|}^{2}.$$

The SNR of the terrestrial link at *U*_2_ is given by

(10) $$\Lambda_{u1u2,ps}=\omega \left(1-\rho \right)\eta_{r}{\left|g_{ru1}\right|}^{2}{\left|g_{u1u2}\right|}^{2}.$$

### 2.2. TS-D2D Scheme

**Figure 2:**
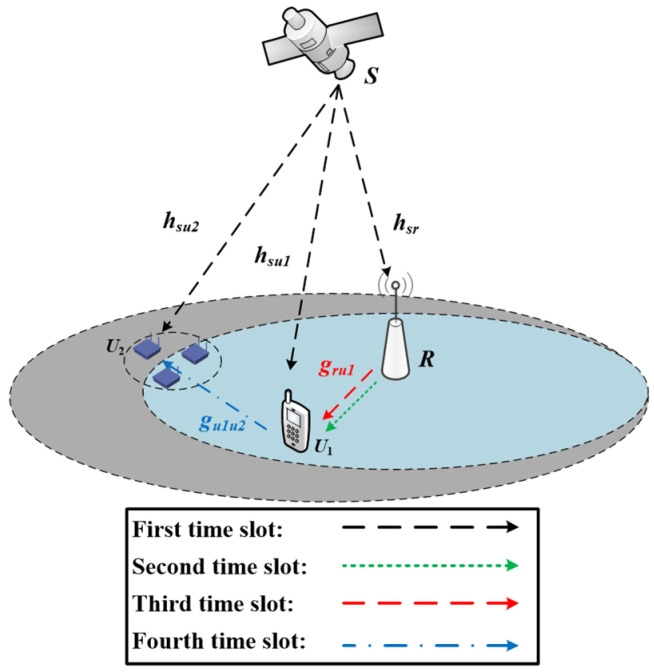
Illustration of the TS-D2D-based HSTN model.

In the TS-D2D scheme, which is shown in Figure 2, we assume that *T* denotes the duration of the entire transmission process, which is divided into four orthogonal phases.

During the first time slot, with a time duration of $\left(1-\tau \right)T/3$, $\tau \in \left(0,1\right)$, the satellite *S* broadcasts its signal $\sqrt{P_{s}}x_{s}$ to the terrestrial nodes. Therefore, the received symbols at the terrestrial nodes can be, respectively, given by

(11) $$y_{si,ts}=\sqrt{P_{s}}h_{si}x_{s}+n_{si}.$$

During the second time slot, with a time duration of $\left(1-\tau \right)T/3$, *R* amplifies and forwards $y_{sr,ts}$ to *U*_1_, and the received symbol will be

(12) $$y_{ru1,ts}=\sqrt{P_{r}}g_{ru1}\frac{y_{sr,ts}}{\sqrt{{\left|y_{sr,ts}\right|}^{2}}}+n_{ru1}.$$

The SNR of satellite–terrestrial direct link at *U*_1_ is given by

(13) $$\Lambda_{su1,ts}=\eta_{s}{\left|h_{su1}\right|}^{2}.$$

The SNR of terrestrial relay link at *U*_1_ is given by

(14) $$\Lambda_{sru1,ts}=\frac{\Lambda_{sr,ts}\Lambda_{ru1,ts}}{\Lambda_{sr,ts}+\Lambda_{ru1,ts}+1},$$

where $\Lambda_{sr,ts}=\eta_{s}{\left|h_{sr}\right|}^{2}$, $\Lambda_{ru1,ts}=\eta_{r}{\left|g_{ru1}\right|}^{2}$.

During the third time slot, with a time duration of τT, *U*_1_ harvests all the energy from the RF signal of *R*. The harvested energy at *U*_1_ can be given by

(15) $$E_{u1,ts}=\omega \tau TP_{r}{\left|g_{ru1}\right|}^{2}.$$

During the fourth time slot, with a time duration of $\left(1-\tau \right)T/3$, *U*_1_ decodes and forwards the signal $x_{u1,ts}$ to *U*_2_ with the harvested energy. The transmit power at *U*_1_ will be given by

(16) $$P_{u1,ts}=\frac{3E_{u1,ts}}{\left(1-\tau \right)T}=\frac{3\omega \tau P_{r}{\left|g_{ru1}\right|}^{2}}{1-\tau }.$$

Thus, the symbol received at *U*_2_ is given by

(17) $$y_{u1u2,ts}=\sqrt{\frac{3\omega \tau P_{r}{\left|g_{ru1}\right|}^{2}}{1-\tau }}g_{u1u2}x_{u1,ts}+n_{u1u2}.$$

The SNR of the satellite–terrestrial direct link at *U*_2_ is given by

(18) $$\Lambda_{su2,ts}=\eta_{s}{\left|h_{su2}\right|}^{2}.$$

The SNR of the terrestrial link at *U*_2_ is given by

(19) $$\Lambda_{u1u2,ts}=\frac{3\omega \tau \eta_{r}{\left|g_{ru1}\right|}^{2}{\left|g_{u1u2}\right|}^{2}}{1-\tau }.$$

### 2.3. Fading Model

We assume that the channels between *S* and the terrestrial nodes undergo independent shadowed Rician fading with the following PDF:

(20) $$f_{{\left|h_{si}\right|}^{2}}(x)=\alpha_{i}{e^{-\beta_{i}x}}_{1}F_{1}(m_{si};1;\delta_{i}x),x\geq 0,$$

where $\alpha_{i}={{\left(2b_{si}m_{si}/\left(2b_{si}m_{si}+\Omega_{si}\right)\right)}^{m_{si}}/2b}_{si}$, $\beta_{i}=(1/2b_{si})$, $\delta_{i}=\Omega_{si}/\left({4b_{si}}^{2}m_{si}+b_{si}\Omega_{si}\right)$; $\Omega_{si}$ denotes the average power of the line of sight (LOS) component, $2b_{si}$ denotes the average power of the multipath component, $m_{si}$ denotes the Nakagami parameter, and F11(msi;1;δix) denotes the confluent hypergeometric function. For convenience, the fading coefficient $m_{si}$ is assumed to be an integer. Then, the the PDF of $\Lambda_{si}$ can be rewritten as

(21) $$f_{\Lambda_{si}}\left(x\right)=\alpha_{i}\sum_{\kappa =0}^{m_{si}-1}\frac{{\left(-1\right)}^{\kappa }{\left(1-m_{si}\right)}_{\kappa }\delta_{i}^{\kappa }}{{\left(\eta_{s}\right)}^{\kappa +1}{\left(\kappa !\right)}^{2}}x^{\kappa }e^{-\left(\frac{\beta_{i}-\delta_{i}}{\eta_{s}}\right)x},$$

where ${\left(\cdot \right)}_{\kappa }$ is the Pochhammer symbol. The corresponding CDF is obtained as follows:

(22) $$F_{\Lambda_{si}}\left(x\right)=1-\alpha_{i}\sum_{\kappa =0}^{m_{si}-1}\frac{{\left(-1\right)}^{\kappa }{\left(1-m_{si}\right)}_{\kappa }\delta_{i}^{\kappa }}{{\left(\eta_{s}\right)}^{\kappa +1}{\left(\kappa !\right)}^{2}}\sum_{p=0}^{\kappa }\frac{\kappa !}{p!}{\left(\frac{\beta_{i}-\delta_{i}}{\eta_{s}}\right)}^{-\left(\kappa +1-p\right)}x^{p}e^{-\left(\frac{\beta_{i}-\delta_{i}}{\eta_{s}}\right)x}.$$

We assume that the channels among terrestrial nodes undergo independent Nakagami-*m* fading. Then, the PDF of $\Lambda_{j}$ can be presented by

(23) $$f_{\Lambda_{j}}(x)={\left(\frac{m_{j}}{\Omega_{j}\eta_{r}}\right)}^{m_{j}}\frac{x^{m_{j}-1}}{\Gamma \left(m_{j}\right)}e^{-\frac{m_{j}x}{\Omega_{j}\eta_{r}}},$$

where $j\in \left\{ru1,u1u2\right\}$, $m_{j}$ denotes the fading coefficient, $\Omega_{j}$ denotes the average power, and $\Gamma \left(\cdot \right)$ is the gamma function. The corresponding CDF is obtained as follows:

(24) $$F_{\Lambda_{j}}(x)=\frac{1}{\Gamma \left(m_{j}\right)}\gamma \left(m_{j},\frac{m_{j}x}{\Omega_{j}\eta_{r}}\right),$$

where γ(⋅,⋅) is the incomplete gamma function.

## 3. Performance Analysis

In this section, the closed-form expressions for the OP of the proposed network for PS-D2D and TS-D2D schemes are obtained, respectively. Specifically, after deriving the OP of the satellite–terrestrial direct link and the terrestrial relay link, respectively, maximal ratio combining (MRC) is employed at the UT and IoT device to obtain their corresponding OP.

### 3.1. PS-D2D Scheme

The OP of the UT and IoT device for the PS-D2D scheme are derived in the following, respectively.

#### 3.1.1. Outage Probability of U_1_

Firstly, we assume that the target rate at *U*_1_ is $R_{p}$ with only the satellite–terrestrial direct link. Thus, the OP of *U*_1_ for the satellite–terrestrial direct link is as follows:

(25) $$P_{\mathrm{out},\mathrm{PS}}^{\mathrm{su}1}(R_{p})=\mathrm{Pr}\left[\log_{2}(1+\Lambda_{su1,ps})<R_{p}\right]=F_{\Lambda_{su1,ps}}({\gamma^{\prime }}_{p}),$$

where ${\gamma^{\prime }}_{p}=2^{R_{p}}-1$. Substituting Equation (22) into Equation (25), we can compute the OP of *U*_1_ for the satellite–terrestrial direct link.

Secondly, we assume that the target rate at *U*_1_ is $R_{p}$ with only the terrestrial relay link. Thus, the OP of *U*_1_ for the terrestrial relay link is as follows:

(26) $$P_{\mathrm{out},\mathrm{PS}}^{\mathrm{sru}1}(R_{p})=\mathrm{Pr}\left[\frac{1}{3}\log_{2}(1+\Lambda_{sru1,ps})<R_{p}\right]=F_{\Lambda_{sru1,ps}}(\gamma_{p}),$$

where $\gamma_{p}=2^{3R_{p}}-1$, and 1/3 results from the three time slots to complete the transmission process from *S* to *U*_1_. Substituting Equation (6) into Equation (26), we can present the CDF of $\Lambda_{sru1,ps}$ as follows:

(27) $$F_{\Lambda_{sru1,ps}}(x)=\mathrm{Pr}\left[\Lambda_{sr,ps}<\frac{x\left(\rho^{\prime }\Lambda_{ru1,ps}+1\right)}{\rho^{\prime }\Lambda_{ru1,ps}-x}\right]=1-\int_{\frac{x}{\rho^{\prime }}}^{\infty }{\bar{F}}_{\Lambda_{sr,ps}}\left(\frac{\rho^{\prime }xy+x}{\rho^{\prime }y-x}\right)f_{\Lambda_{ru1,ps}}(y)dy,$$

where ${\bar{F}}_{\Lambda_{sr,ps}}(\cdot )=1-F_{\Lambda_{sr,ps}}(\cdot )$ denotes the complementary CDF of $\Lambda_{sr,ps}$. Substituting Equations (22) and (23) into Equation (27), and simplifying using binomial expansion in reference [22], we can obtain the CDF of $\Lambda_{sru1,ps}$ as follows:

(28) FΛsru1,ps(x)=1−2αr∑κ=0msr−1∑l=0κ∑m=0l∑g=0m+mcb−1(−1)κ(1−msr)κδrκ(ηs)κ+1κ!l!(βr−δrηs)−(κ+1−l)            ×(mru1Ωru1ηr)mru11Γ(mru1)xm+mcb−1−gρ′mru1lmm+mru1−1g            ×e−βr−δrηs+mru1ρ′Ωru1ηrxβr−δrηsΩru1ηr(x2+ρ′x)mru1g−l+12xl            ×Kg−l+1(2ρ′βr−δrηsmru1(x2+ρ′x)ρ′Ωru1ηr),

where $K_{v}(\cdot )$ is the modified Bessel function of the second kind. After substituting Equation (28) into Equation (26) with the threshold data rate $\gamma_{p}$, we can obtain the OP of *U*_1_ for the terrestrial relay link.

Thirdly, utilizing Equations (5) and (6) for MRC, we have the OP of *U*_1_ for PS-D2D scheme as follows:

(29) $$\begin{array}{cc} P_{\mathrm{out},\mathrm{PS}}^{u1}(R_{p}) & =\mathrm{Pr}\left[\log_{2}(1+\Lambda_{su1,ps})<R_{p},\frac{1}{3}\log_{2}(\Lambda_{su1,ps}+\Lambda_{sru1,ps})<R_{p}\right]\\ & =\mathrm{Pr}\left[\Lambda_{su1,ps}<{\gamma^{\prime }}_{p},\Lambda_{su1,ps}+\Lambda_{sru1,ps}<\gamma_{p}\right]\\ & =\mathrm{Pr}\left[\Lambda_{su1,ps}<\min(\gamma_{p}-\Lambda_{sru1,ps},{\gamma^{\prime }}_{p})\right]\\ & =\phi_{1}+\phi_{2}, \end{array}$$

where $\phi_{1}$ denotes the probability of case 1, and $\phi_{2}$ denotes the probability of case 2. The probability of case 1 is given by

(30) $$\phi_{1}=\mathrm{Pr}\left[\Lambda_{su1,ps}<{\gamma^{\prime }}_{p},{\gamma^{\prime }}_{p}<\gamma_{p}-\Lambda_{sru1,ps}\right]=F_{\Lambda_{su1,ps}}({\gamma^{\prime }}_{p})F_{\Lambda_{sru1,ps}}(\gamma_{p}-{\gamma^{\prime }}_{p}).$$

Substituting Equations (25) and (26) into Equation (30), we can obtain $\phi_{1}$. The probability of case 2 is given by

(31) $$\begin{array}{l} \phi_{2}=\mathrm{Pr}\left[\Lambda_{su1,ps}<\gamma_{p}-\Lambda_{sru1,ps},{\gamma^{\prime }}_{p}\geq \gamma_{p}-\Lambda_{sru1,ps}\right]\\ \;\;\;\;=\mathrm{Pr}\left[\Lambda_{su1,ps}<\gamma_{p}-\Lambda_{sru1,ps},\Lambda_{sru1,ps}\geq \gamma_{p}-{\gamma^{\prime }}_{p}\right]\\ \;\;\;\;=\int_{0}^{\gamma_{p}}\int_{\gamma_{p}-{\gamma^{\prime }}_{p}}^{\gamma_{p}-y}f_{\Lambda_{sru1,ps}}(x)f_{\Lambda_{su1,ps}}(y)dxdy\\ \;\;\;\;=\int_{0}^{\gamma_{p}}F_{\Lambda_{sru1,ps}}(\gamma_{p}-y)f_{\Lambda_{su1,ps}}(y)dy-\int_{0}^{\gamma_{p}}F_{\Lambda_{sru1,ps}}(\gamma_{p}-{\gamma^{\prime }}_{p})f_{\Lambda_{su1,ps}}(y)dy. \end{array}$$

Making use of the *L*-step staircase approximation approach in reference [23] for the included triangular integral region in Equation (31), we can rewrite the probability of case 2 as

(32) $$\begin{array}{c} \phi_{2}\approx \sum_{i=0}^{L-1}\left\{F_{\Lambda_{su1,ps}}\left(\frac{i+1}{L}\gamma_{p}\right)-F_{\Lambda_{su1,ps}}\left(\frac{i}{L}\gamma_{p}\right)\right\}\\ \;\;\;\;\;\;\;\;\;\;\;\;\;\;\;\;\;\;\;\;\;\;\;\;\;\;\;\;\;\;\;\;\;\;\times F_{\Lambda_{sru1,ps}}\left(\frac{L-i}{L}\gamma_{p}\right)-F_{\Lambda_{sru1,ps}}(\gamma_{p}-{\gamma^{\prime }}_{p})F_{\Lambda_{su1,ps}}(\gamma_{p}). \end{array}$$

Substituting Equations (25) and (26) into Equation (32), we can obtain $\phi_{2}$. Then, substituting Equations (30) and (32) into Equation (29), we can obtain the OP of *U*_1_ for the PS-D2D scheme.

#### 3.1.2. Outage Probability of U_2_

Firstly, we assume that the target rate at *U*_2_ is $R_{p}$ with only the satellite–terrestrial direct link. Thus, the OP of *U*_2_ for the satellite–terrestrial direct link is as follows:

(33) $$P_{\mathrm{out},\mathrm{PS}}^{\mathrm{su}2}(R_{p})=\mathrm{Pr}\left[\log_{2}(1+\Lambda_{su2,ps})<R_{p}\right]=F_{\Lambda_{su2,ps}}({\gamma^{\prime }}_{p}).$$

Substituting Equation (22) into Equation (33), we can compute the OP of *U*_2_ for the satellite–terrestrial direct link.

Secondly, considering that the terrestrial relay link of *U*_2_ originates from *U*_1_ with the SNR of $\Lambda_{u1u2,ps}$ in Equation (10), the OP of *U*_2_ for the terrestrial relay link is the CDF of $\Lambda_{u1u2,ps}$. Making use of the PDF of the gamma random variable in reference [24] for Equation (10), we can obtain the PDF of $\Lambda_{u1u2,ps}$ as follows:

(34) $$\begin{array}{c} f_{\Lambda_{u1u2,ps}}(x)=\int_{0}^{\infty }\frac{1}{\omega (1-\rho )y}f_{\eta_{r}{\left|g_{ru1}\right|}^{2}}\left(\frac{x}{\omega y-\omega y\rho }\right)f_{{\left|g_{u1u2}\right|}^{2}}(y)dy\\ \;\;\;\;\;\;\;\;\;\;\;\;\;\;\;\;\;\;\;\;\;\;\;\;\;\;\;\;\;\;\;\;=\frac{2{\left(\chi_{1}\right)}^{\frac{m_{ru1}+m_{u1u2}}{2}}}{\Gamma (m_{ru1})\Gamma (m_{u1u2})}x^{\frac{m_{ru1}+m_{u1u2}}{2}-1}K_{m_{u1u2}-m_{ru1}}(2\sqrt{\chi_{1}x}), \end{array}$$

where $\chi_{1}=\frac{m_{ru1}m_{u1u2}}{\omega (1-\rho )\Omega_{ru1}\Omega_{u1u2}\eta_{r}}$. Making use of Meijer’s G-function in reference [25], we can rewrite the PDF of $\Lambda_{u1u2,ps}$ as

(35) $$f_{\Lambda_{u1u2,ps}}(x)=\frac{\chi_{1}}{\Gamma (m_{ru1})\Gamma (m_{u1u2})}G_{0,2}^{2,0}\left[\chi_{1}x\left|\begin{array}{l} -\\ \frac{m_{u1u2}-m_{ru1}}{2},-\frac{m_{u1u2}-m_{ru1}}{2} \end{array}\right.\right],$$

where $G_{p,q}^{m,n}\left[x\left|\begin{array}{l} a_{1},\cdot \cdot \cdot ,a_{p}\\ b_{1},\cdot \cdot \cdot ,b_{q} \end{array}\right.\right]$ is the Meijer’s G-function. Making use of the integral formula of Meijer’s G-function in reference [26], we can obtain the corresponding CDF of $\Lambda_{u1u22.ps}$ as follows:

(36) $$F_{\Lambda_{u1u2,ps}}(x)=\frac{\chi_{1}x}{\Gamma (m_{ru1})\Gamma (m_{u1u2})}G_{1,3}^{2,1}\left[\chi_{1}x\left|\begin{array}{l} 0,\\ \frac{m_{u1u2}-m_{ru1}}{2},-\frac{m_{u1u2}-m_{ru1}}{2},-1 \end{array}\right.\right].$$

Thirdly, utilizing Equations (9) and (10) for MRC, we have the OP of *U*_2_ for the PS-D2D scheme as follows:

(37) $$\begin{array}{cc} P_{\mathrm{out},\mathrm{PS}}^{u2}(R_{p}) & =\mathrm{Pr}\left[\log_{2}(1+\Lambda_{su2,ps})<R_{p},\frac{1}{3}\log_{2}(\Lambda_{su2,ps}+\Lambda_{u1u2,ps})<R_{p}\right]\\ & =\mathrm{Pr}\left[\Lambda_{su2,ps}<{\gamma^{\prime }}_{p},\Lambda_{su2,ps}+\Lambda_{u1u2,ps}<\gamma_{p}\right]\\ & =\mathrm{Pr}\left[\Lambda_{su2,ps}<\min(\gamma_{p}-\Lambda_{u1u2,ps},{\gamma^{\prime }}_{p})\right]\\ & =\phi_{3}+\phi_{4}, \end{array}$$

where $\phi_{3}$ denotes the probability of case 3, and $\phi_{4}$ denotes the probability of case 4. The probability of case 3 is as follows:

(38) $$\phi_{3}=\mathrm{Pr}\left[\Lambda_{su2,ps}<{\gamma^{\prime }}_{p},{\gamma^{\prime }}_{p}<\gamma_{p}-\Lambda_{sru2,ps}\right]=F_{\Lambda_{su2,ps}}({\gamma^{\prime }}_{p})F_{\Lambda_{u1u2,ps}}(\gamma_{p}-{\gamma^{\prime }}_{p}).$$

Substituting Equations (33) and (36) into Equation (38), we can obtain $\phi_{3}$. The probability of case 4 is as follows:

(39) $$\begin{array}{cc} \phi_{4} & =\mathrm{Pr}\left[\Lambda_{su2,ps}<\gamma_{p}-\Lambda_{u1u2,ps},{\gamma^{\prime }}_{p}\geq \gamma_{p}-\Lambda_{u1u2,ps}\right]\\ & =\mathrm{Pr}\left[\Lambda_{su2,ps}<\gamma_{p}-\Lambda_{u1u2,ps},\Lambda_{u1u2,ps}\geq \gamma_{p}-{\gamma^{\prime }}_{p}\right]\\ & =\int_{0}^{\gamma_{p}}\int_{\gamma_{p}-{\gamma^{\prime }}_{p}}^{\gamma_{p}-y}f_{\Lambda_{u1u2,ps}}(x)f_{\Lambda_{su2,ps}}(y)dxdy\\ & =\int_{0}^{\gamma_{p}}F_{\Lambda_{u1u2,ps}}(\gamma_{p}-y)f_{\Lambda_{su2,ps}}(y)dy-\int_{0}^{\gamma_{p}}F_{\Lambda_{u1u2,ps}}(\gamma_{p}-{\gamma^{\prime }}_{p})f_{\Lambda_{su2,ps}}(y)dy. \end{array}$$

Adopting the *L*-step staircase approximation approach, we can rewrite the probability of case 4 as

(40) $$\begin{array}{c} \phi_{4}\approx \sum_{i=0}^{L-1}\left\{F_{\Lambda_{su2,ps}}\left(\frac{i+1}{L}\gamma_{p}\right)-F_{\Lambda_{su2,ps}}\left(\frac{i}{L}\gamma_{p}\right)\right\}\\ \;\;\;\;\;\;\;\;\;\;\;\;\;\;\;\;\;\;\;\;\;\;\;\;\;\;\;\;\;\;\;\;\;\;\;\times F_{\Lambda_{u1u2,ps}}\left(\frac{L-i}{L}\gamma_{p}\right)-F_{\Lambda_{u1u2,ps}}(\gamma_{p}-{\gamma^{\prime }}_{p})F_{\Lambda_{su2,ps}}(\gamma_{p}). \end{array}$$

Substituting Equations (33) and (36) into Equation (40), we can obtain $\phi_{4}$. Then, substituting Equations (38) and (40) into Equation (37), we can obtain the OP of *U*_2_ for the PS-D2D scheme.

### 3.2. TS-D2D Scheme

In what follows, the OP of the UT and IoT device for the TS-D2D scheme are derived in the following, respectively.

#### 3.2.1. Outage Probability of U_1_

Firstly, we assume that the target rate at *U*_1_ is $R_{p}$ with only the satellite–terrestrial direct link. Thus, the OP of *U*_1_ for the satellite–terrestrial direct link is as follows:

(41) $$P_{\mathrm{out},\mathrm{TS}}^{\mathrm{su}1}(R_{p})=\mathrm{Pr}\left[\log_{2}(1+\Lambda_{su1,ts})<R_{p}\right]=F_{\Lambda_{su1,ts}}({\gamma^{\prime }}_{p}).$$

Substituting Equation (22) into Equation (41), we can compute the OP of *U*_1_ for the satellite–terrestrial direct link.

Secondly, we assume that the target rate at *U*_1_ is $R_{p}$ with only the terrestrial relay link. Thus, the OP of *U*_1_ for the terrestrial relay link is as follows:

(42) $$P_{\mathrm{out},\mathrm{TS}}^{\mathrm{sru}1}(R_{p})=\mathrm{Pr}\left[\frac{1-\tau }{3}\log_{2}(1+\Lambda_{sru1,ts})<R_{p}\right]=F_{\Lambda_{sru1,ts}}(\gamma_{p,ts}),$$

where $\gamma_{p,ts}=2^{\left(3R_{p}\right)/\left(1-\tau \right)}-1$. Substituting Equation (14) into Equation (42), we can present the CDF of $\Lambda_{sru1,ts}$ as follows:

(43) $$F_{\Lambda_{sru1,ts}}(x)=\mathrm{Pr}\left[\Lambda_{sr,ts}<\frac{x\left(\Lambda_{ru1,ts}+1\right)}{\Lambda_{ru1,ts}-x}\right]=1-\int_{x}^{\infty }{\bar{F}}_{\Lambda_{sr,ts}}\left(\frac{xy+x}{y-x}\right)f_{\Lambda_{ru1,ts}}(y)dy,$$

where ${\bar{F}}_{\Lambda_{sr,ts}}(\cdot )=1-F_{\Lambda_{sr,ts}}(\cdot )$ denotes the complementary CDF of $\Lambda_{sr,ts}$. Substituting Equations (22) and (23) into Equation (43), and simplifying using binomial expansion, we can obtain the CDF of $\Lambda_{sru1,ts}$ as follows:

(44) FΛsru1,ts(x)=1−2αr∑κ=0msr−1∑l=0κ∑m=0l∑g=0m+mcb−1(−1)κ(1−msr)κδrκ(ηs)κ+1κ!l!(βr−δrηs)−(κ+1−l)                ×(mru1Ωru1ηr)mru1xm+mcb−1−gΓ(mru1)(lm)(m+mru1−1g)                ×e−βr−δrηs+mru1Ωru1ηrxβr−δrηsΩru1ηr(x2+x)mru1g−l+12xl                ×Kg−l+1(2βr−δrηsmru1(x2+x)Ωru1ηr).

After substituting Equation (44) into Equation (42) with the threshold data rate $\gamma_{p,ts}$, we can obtain the OP of *U*_1_ for the terrestrial relay link.

Thirdly, utilizing Equations (13) and (14) for MRC, we have the OP of *U*_1_ for TS-D2D scheme as follows:

(45) $$\begin{array}{cc} P_{\mathrm{out},\mathrm{TS}}^{u1}(R_{p}) & =\mathrm{Pr}\left[\log_{2}(1+\Lambda_{su1,ts})<R_{p},\frac{1-\tau }{3}\log_{2}(\Lambda_{su1,ts}+\Lambda_{sru1,ts})<R_{p}\right]\\ & =\mathrm{Pr}\left[\Lambda_{su1,ts}<{\gamma^{\prime }}_{p},\Lambda_{su1,ts}+\Lambda_{sru1,ts}<\gamma_{p,ts}\right]\\ & =\mathrm{Pr}\left[\Lambda_{su1,ts}<\min(\gamma_{p,ts}-\Lambda_{sru1,ts},{\gamma^{\prime }}_{p})\right]\\ & =\varphi_{1}+\varphi_{2}, \end{array}$$

where $\varphi_{1}$ denotes the probability of case 1, and $\varphi_{2}$ denotes the probability of case 2. The probability of case 1 is given by

(46) $$\varphi_{1}=\mathrm{Pr}\left[\Lambda_{su1,ts}<{\gamma^{\prime }}_{p},{\gamma^{\prime }}_{p}<\gamma_{p,ts}-\Lambda_{sru1,ts}\right]=F_{\Lambda_{su1,ts}}({\gamma^{\prime }}_{p})F_{\Lambda_{sru1,ts}}(\gamma_{p,ts}-{\gamma^{\prime }}_{p}).$$

Substituting Equations (41) and (42) into Equation (46), we can obtain $\varphi_{1}$. The probability of case 2 is given by

(47) $$\begin{array}{cc} \varphi_{2} & =\mathrm{Pr}\left[\Lambda_{su1,ts}<\gamma_{p,ts}-\Lambda_{sru1,ts},{\gamma^{\prime }}_{p}\geq \gamma_{p,ts}-\Lambda_{sru1,ts}\right]\\ & =\mathrm{Pr}\left[\Lambda_{su1,ts}<\gamma_{p,ts}-\Lambda_{sru1,ts},\Lambda_{sru1,ts}\geq \gamma_{p,ts}-{\gamma^{\prime }}_{p}\right]\\ & =\int_{0}^{\gamma_{p,ts}}\int_{\gamma_{p,ts}-{\gamma^{\prime }}_{p}}^{\gamma_{p,ts}-y}f_{\Lambda_{sru1,ts}}(x)f_{\Lambda_{su1,ts}}(y)dxdy\\ & =\int_{0}^{\gamma_{p,ts}}F_{\Lambda_{sru1,ts}}(\gamma_{p,ts}-y)f_{\Lambda_{su1,ts}}(y)dy-\int_{0}^{\gamma_{p,ts}}F_{\Lambda_{sru1,ts}}(\gamma_{p,ts}-{\gamma^{\prime }}_{p})f_{\Lambda_{su1,ts}}(y)dy. \end{array}$$

Adopting the *L*-step staircase approximation approach, we can rewrite the probability of case 2 as

(48) $$\begin{array}{c} \varphi_{2}\approx \sum_{i=0}^{L-1}\left\{F_{\Lambda_{su1,ts}}\left(\frac{i+1}{L}\gamma_{p,ts}\right)-F_{\Lambda_{su1,ts}}\left(\frac{i}{L}\gamma_{p,ts}\right)\right\}\\ \;\;\;\;\;\;\;\;\;\;\;\;\;\;\;\;\;\;\;\;\;\;\;\;\;\;\;\;\;\;\;\;\;\;\;\times F_{\Lambda_{sru1,ts}}\left(\frac{L-i}{L}\gamma_{p,ts}\right)-F_{\Lambda_{sru1,ts}}(\gamma_{p,ts}-{\gamma^{\prime }}_{p})F_{\Lambda_{su1,ts}}(\gamma_{p,ts}). \end{array}$$

Substituting Equations (41) and (42) into Equation (48), we can obtain $\varphi_{2}$. Then, substituting Equations (46) and (48) into Equation (45), we can obtain the OP of *U*_1_ for the TS-D2D scheme.

#### 3.2.2. Outage Probability of U_2_

Firstly, we assume that the target rate at *U*_2_ is $R_{p}$ with only the satellite–terrestrial direct link. Thus, the OP of *U*_2_ for the satellite–terrestrial direct link is as follows:

(49) $$P_{\mathrm{out},\mathrm{TS}}^{\mathrm{su}2}(R_{p})=\mathrm{Pr}\left[\log_{2}(1+\Lambda_{su2,ts})<R_{p}\right]=F_{\Lambda_{su2,ts}}({\gamma^{\prime }}_{p}).$$

Substituting Equation (22) into Equation (49), we can compute the OP of *U*_2_ for the satellite–terrestrial direct link.

Secondly, considering that the terrestrial relay link of *U*_2_ originates from *U*_1_ with the SNR of $\Lambda_{u1u2,ts}$ in Equation (19), the OP of *U*_2_ for the terrestrial relay link is the CDF of $\Lambda_{u1u2,ts}$. Making use of the PDF of the gamma random variable for Equation (19), we can obtain the PDF of $\Lambda_{u1u2,ts}$ as follows:

(50) $$\begin{array}{cc} f_{\Lambda_{u1u2,ts}}(x) & =\int_{0}^{\infty }\frac{1-\tau }{3\omega \tau y}f_{\eta_{r}{\left|g_{ru1}\right|}^{2}}\left(\frac{x-x\tau }{3\omega \tau y}\right)f_{{\left|g_{u1u2}\right|}^{2}}(y)dy\\ & =\frac{2{\left(\chi_{2}\right)}^{\frac{m_{ru1}+m_{u1u2}}{2}}}{\Gamma (m_{ru1})\Gamma (m_{u1u2})}x^{\frac{m_{ru1}+m_{u1u2}}{2}-1}K_{m_{u1u2}-m_{ru1}}(2\sqrt{\chi_{2}x})\\ & =\frac{\chi_{2}}{\Gamma (m_{ru1})\Gamma (m_{u1u2})}G_{0,2}^{2,0}\left[\chi_{2}x\left|\begin{array}{l} -\\ \frac{m_{u1u2}-m_{ru1}}{2},-\frac{m_{u1u2}-m_{ru1}}{2} \end{array}\right.\right], \end{array}$$

where $\chi_{2}=\frac{\left(1-\tau \right)m_{ru1}m_{u1u2}}{3\omega \tau \Omega_{ru1}\Omega_{u1u2}\eta_{r}}$. The corresponding CDF of $\Lambda_{u1u2,ts}$ can be presented by

(51) $$F_{\Lambda_{u1u2,ts}}(x)=\frac{\chi_{2}x}{\Gamma (m_{ru1})\Gamma (m_{u1u2})}G_{1,3}^{2,1}\left[\chi_{2}x\left|\begin{array}{l} 0,\\ \frac{m_{u1u2}-m_{ru1}}{2},-\frac{m_{u1u2}-m_{ru1}}{2},-1 \end{array}\right.\right].$$

Thirdly, utilizing Equations (18) and (19) for MRC, we have the OP of *U*_2_ for the TS-D2D scheme as follows:

(52) $$\begin{array}{cc} P_{\mathrm{out},\mathrm{TS}}^{u2}(R_{p}) & =\mathrm{Pr}\left[\log_{2}(1+\Lambda_{su2,ts})<R_{p},\frac{1-\tau }{3}\log_{2}(\Lambda_{su2,ts}+\Lambda_{u1u2,ts})<R_{p}\right]\\ & =\mathrm{Pr}\left[\Lambda_{su2,ts}<{\gamma^{\prime }}_{p},\Lambda_{su2,ts}+\Lambda_{u1u2,ts}<\gamma_{p,ts}\right]\\ & =\mathrm{Pr}\left[\Lambda_{su2,ts}<\min(\gamma_{p,ts}-\Lambda_{u1u2,ts},{\gamma^{\prime }}_{p})\right]\\ & =\varphi_{3}+\varphi_{4}, \end{array}$$

where $\varphi_{3}$ denotes the probability of case 3, and $\varphi_{4}$ denotes the probability of case 4. The probability of case 3 is as follows:

(53) $$\varphi_{3}=\mathrm{Pr}\left[\Lambda_{su2,ts}<{\gamma^{\prime }}_{p},{\gamma^{\prime }}_{p}<\gamma_{p,ts}-\Lambda_{sru2,ts}\right]=F_{\Lambda_{su2,ts}}({\gamma^{\prime }}_{p})F_{\Lambda_{u1u2,ts}}(\gamma_{p,ts}-{\gamma^{\prime }}_{p}).$$

Substituting Equations (49) and (51) into Equation (53), we can obtain $\varphi_{3}$. The probability of case 4 is as follows:

(54) $$\begin{array}{cc} \varphi_{4} & =\mathrm{Pr}\left[\Lambda_{su2,ts}<\gamma_{p,ts}-\Lambda_{u1u2,ts},{\gamma^{\prime }}_{p}\geq \gamma_{p,ts}-\Lambda_{u1u2,ts}\right]\\ & =\mathrm{Pr}\left[\Lambda_{su2,ts}<\gamma_{p,ts}-\Lambda_{u1u2,ts},\Lambda_{u1u2,ts}\geq \gamma_{p,ts}-{\gamma^{\prime }}_{p}\right]\\ & =\int_{0}^{\gamma_{p,ts}}\int_{\gamma_{p,ts}-{\gamma^{\prime }}_{p}}^{\gamma_{p,ts}-y}f_{\Lambda_{u1u2,ts}}(x)f_{\Lambda_{su2,ts}}(y)dxdy\\ & =\int_{0}^{\gamma_{p,ts}}F_{\Lambda_{u1u2,ts}}(\gamma_{p,ts}-y)f_{\Lambda_{su2,ts}}(y)dy-\int_{0}^{\gamma_{p,ts}}F_{\Lambda_{u1u2,ts}}(\gamma_{p,ts}-{\gamma^{\prime }}_{p})f_{\Lambda_{su2,ts}}(y)dy. \end{array}$$

Adopting the *L*-step staircase approximation approach, we can rewrite the probability of case 4 as

(55) $$\begin{array}{c} \varphi_{4}\approx \sum_{i=0}^{L-1}\left\{F_{\Lambda_{su2,ts}}\left(\frac{i+1}{L}\gamma_{p,ts}\right)-F_{\Lambda_{su2,ts}}\left(\frac{i}{L}\gamma_{p,ts}\right)\right\}\\ \;\;\;\;\;\;\;\;\;\;\;\;\;\;\;\;\;\;\;\;\;\;\;\;\;\;\;\;\;\;\;\;\;\;\;\;\;\times F_{\Lambda_{u1u2,ts}}\left(\frac{L-i}{L}\gamma_{p,ts}\right)-F_{\Lambda_{u1u2,ts}}(\gamma_{p,ts}-{\gamma^{\prime }}_{p})F_{\Lambda_{su2,ts}}(\gamma_{p,ts}). \end{array}$$

Substituting Equations (49) and (51) into Equation (55), we can obtain $\varphi_{4}$. Then, substituting Equations (53) and (55) into Equation (52), we can obtain the OP of *U*_2_ for the TS-D2D scheme.

## 4. Numerical Results

In this section, we conduct numerical investigations for the proposed PS-D2D-based and TS-D2D-based HSTNs, and validate our analytical OP expressions through Monte Carlo simulations. Unless otherwise explicitly specified, the parameters are set as ω=0.7, $R_{p}=0.5\;\mathrm{bps}/\mathrm{Hz}$, τ=0.1, ${\gamma^{\prime }}_{p}=0.414$, $\gamma_{p}=1.828$, $\gamma_{p,ts}=2.182$, $\Omega_{ru1}=\Omega_{u1u2}=1$, and $m_{u1u2}=1$, and $\eta_{s}=\eta_{r}$ as the SNR. The shadowed Rician fading parameters for the satellite–terrestrial link *S* − *R* are considered under the average shadowing (AS) condition as $m_{sr}=5$, $b_{sr}=0.251$, and $\Omega_{sr}=0.279$ in reference [27]. The shadowed Rician fading parameters for the satellite–terrestrial direct link *S* − *U*_2_ are considered under the heavy shadowing (HS) condition as $m_{su2}=2$, $b_{su2}=0.063$, and $\Omega_{su2}=0.0005$ in reference [27]. To make the relative approximation error negligible, we set L=20 in the *L*-step staircase approximation approach. To verify the proposed analysis models, $10^{5}$ channel realizations of shadowed Rician and Nakagami-*m* fading are generated.

**Figure 3:**
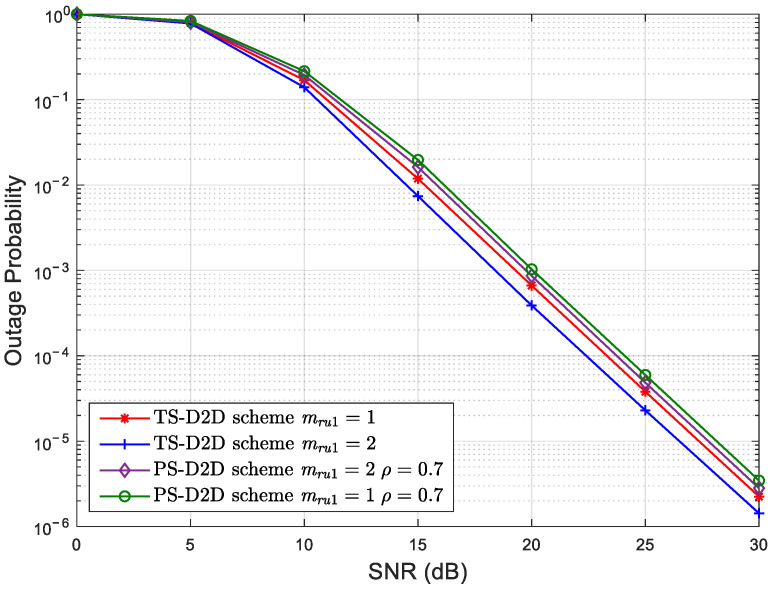
The OP of *U*_1_ versus the SNR with different Nakagami coefficients for the TS-D2D and PS-D2D schemes.

Figure 3 depicts the OP curves of *U*_1_ against the SNR with different Nakagami coefficients for the TS-D2D and PS-D2D schemes with ρ=0.7. Herein, the satellite–terrestrial direct link *S* − *U*_1_ is considered under the AS condition. Even though the threshold data rate of the TS-D2D scheme is slightly higher than that of the PS-D2D scheme, the outage performance of *U*_1_ for the TS-D2D scheme is still better than that for the PS-D2D scheme. This is due to the increased amount of energy harvested and utilized for the ID of the TS-D2D scheme than that for the PS-D2D scheme. Furthermore, the terrestrial relay link achieves a higher outage performance gain for the TS-D2D scheme than that for the PS-D2D scheme.

**Figure 4:**
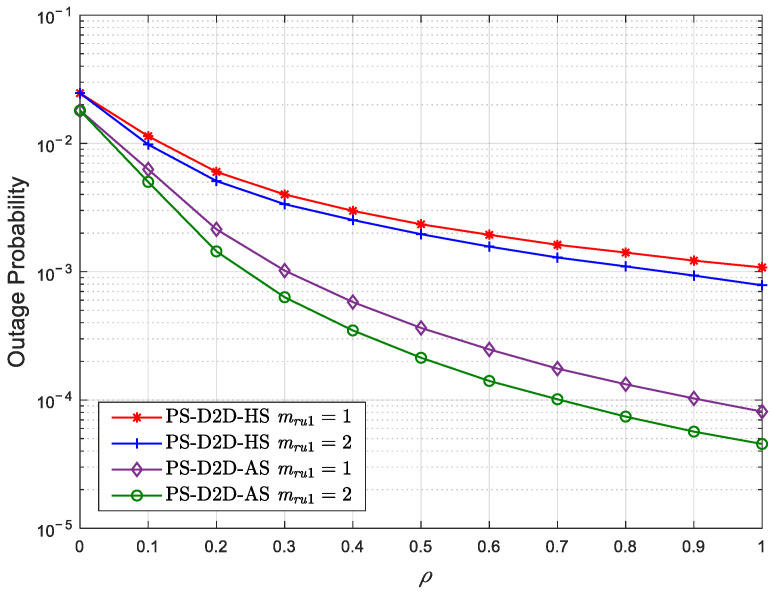
The OP of *U*_1_ versus the PS coefficient with different Nakagami coefficients under HS and AS conditions for the PS-D2D scheme.

Figure 4 illustrates the OP curves of *U*_1_ against the PS coefficient with different Nakagami coefficients under HS and AS conditions for the PS-D2D scheme with $\eta_{s}=23\;\mathrm{dB}$. Herein, the satellite–terrestrial direct link *S* − *U*_1_ is considered under HS and AS conditions, respectively. It can be observed that the outage performance gradually improves as the PS coefficient increases. This is due to the fact that *U*_1_ utilizes more energy for ID. Significantly, *U*_1_ can achieve the optimal outage performance by using all the energy for ID with ρ=1. However, the cooperation from *U*_1_ to *U*_2_ will be ineffective.

**Figure 5:**
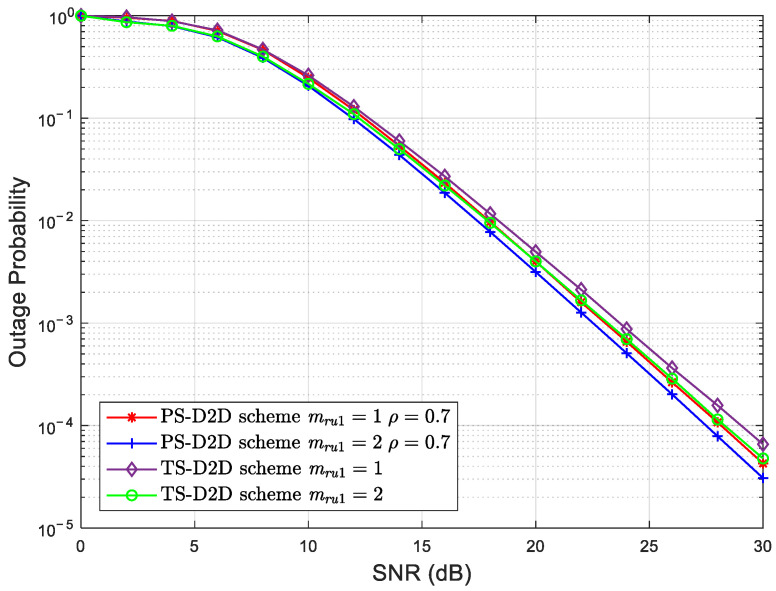
The OP of *U*_2_ versus the SNR with different Nakagami coefficients for the TS-D2D and PS-D2D schemes.

Figure 5 exhibits the OP curves of *U*_2_ against the SNR with different Nakagami coefficients for the TS-D2D and PS-D2D schemes with ρ=0.7. Herein, the satellite–terrestrial direct link *S* − *U*_1_ is considered under the AS condition. Even though the transmit power of the TS-D2D scheme is similar to that of the PS-D2D scheme, the outage performance of *U*_2_ for the PS-D2D scheme is still better than that for the TS-D2D scheme. This is due to the presence of the energy transmission time slot of the TS-D2D scheme, which results in a higher threshold data rate for the TS-D2D scheme than that for the PS-D2D scheme.

**Figure 6:**
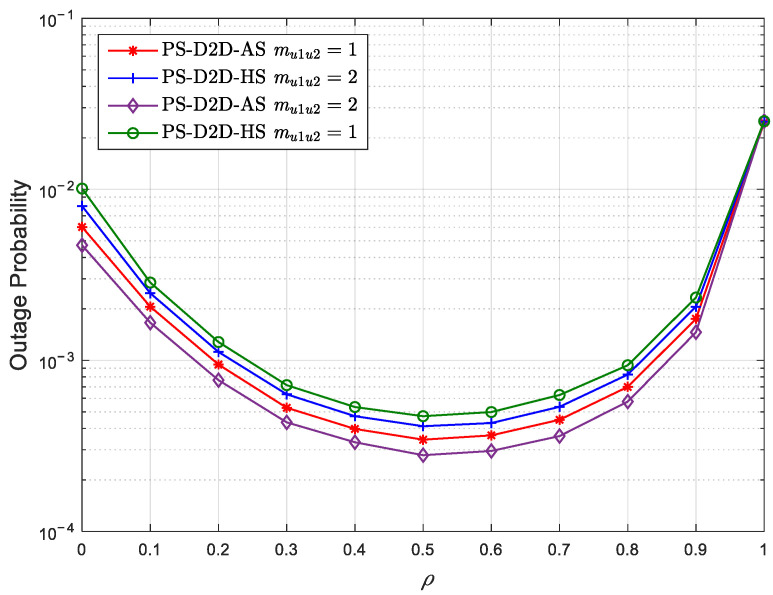
The OP of *U*_2_ versus the PS coefficient with different Nakagami coefficients under HS and AS conditions for the PS-D2D scheme.

Figure 6 presents the OP curves of *U*_2_ against the PS coefficient with different Nakagami coefficients under HS and AS conditions for the PS-D2D scheme with $m_{ru1}=1$ and $\eta_{s}=25\;\mathrm{dB}$. Herein, the satellite–terrestrial direct link *S* − *U*_1_ is considered under HS and AS conditions, respectively. It can be observed that the outage performance gradually increases as the PS coefficient increases from 0 to 0.5 and significantly decreases as the PS coefficient increases from 0.5 to 1. The increase in outage performance from 0 to 0.5 is owing to the fact that the outage performance gain resulting from using more energy for ID is greater than the loss caused by reducing the transmit power. The decrease in outage performance from 0.5 to 1 is due to the fact that the outage performance gain generated by using more energy for ID is less than the loss caused by reducing the transmit power. Significantly, *U*_2_ can achieve the optimal outage performance with ρ=0.5.

## 5. Conclusions

In this paper, we have addressed the HSTNs in which an energy-constrained terrestrial UT cooperates with a shadowed terrestrial IoT device in D2D communication. For this set-up, we have proposed PS-D2D and TS-D2D schemes for the energy-constrained UT to obtain sustainable energy for transmitting information to the shadowed IoT device. We have analyzed the system performance by deriving the closed-form expressions for the OP of both the UT and the IoT device, and have validated our theoretical analyses via Monte Carlo simulations. In the proposed schemes, the UT can utilize the energy harvested from the terrestrial relay to provide a relaying link for the IoT device, thereby improving the outage performance of the IoT device and expanding the terrestrial coverage area of the HSTNs.

## Data Availability

Data are contained within the article.
